# A phenomenological study of online assessment during a pandemic crisis: Insights from Malaysia, Lithuania, and Spain

**DOI:** 10.3389/fpsyg.2022.957896

**Published:** 2022-09-15

**Authors:** Nagaletchimee Annamalai, Antonia Ramírez García, Viktorija Mažeikienė, Marwan Harb Alqaryouti, Radzuwan Ab Rashid, Arulselvi Uthayakumaran

**Affiliations:** ^1^School of Distance Education (English Section), Universiti Sains Malaysia, George Town, Malaysia; ^2^Faculty of Educational Sciences, University of Córdoba, Córdoba, Spain; ^3^Faculty of Human and Social Studies, Mykolas Romeris University, Vilnius, Lithuania; ^4^Department of English Language, Literature and Translation, Zarqa University, Zarqa, Jordan; ^5^Faculty of Languages and Communication, Universiti Sultan Zainal Abidin, Kuala Terengganu, Malaysia; ^6^Centre of Modern Languages, University of Malaysia Pahang, Pahang, Malaysia

**Keywords:** assessment, COVID-19 pandemic, online learning, student experience, phenomenology

## Abstract

Many countries, namely, Malaysia, Lithuania, and Spain, shifted to online assessment during the COVID-19 pandemic. This qualitative case study, which involved 18 undergraduate students from the three countries, was conducted to probe insights into their online assessment experience. Data were interpreted from the perspective of the expectancy-value theory of motivation, which focuses on intrinsic attainment, utility, and cost values. The findings revealed that students were motivated to complete their assessment since they experienced flexibility besides having effective assessment guidelines. The positive experiences were related to intrinsic and attainment values; however, the students were also demotivated when stressed, indicating the high-cost value. Utility value was found to overlap with cost value in this study because students were dissatisfied with the online assessment and expressed less preference for this approach in future. This contributes to our understanding that educators must consider utility values when preparing online assessments. The pedagogical implications of this study revolve around the importance of a checklist, mock exams, alternative assessment (Plan B), and video demos for an effective assessment.

## Introduction

COVID-19 was classified as a global pandemic by WHO in March 2020. UNESCO urged higher education institutions to consider remote teaching and online learning. Henceforth, a sudden change was required without much planning, where teachers and students had to become familiar with various online tools and technologies. At the initial stage of the pandemic, many countries postponed their examinations (Butler-Henderson and Crawford, 2020). Still, as the pandemic continued, many higher education institutions decided on alternative assessment and online examination. This was not a common practice for most students, and the motivation to complete the assessment warranted an investigation. Implementing new approaches, methods, and technological tools can be challenging and since the disruption in the assessment practice is a global phenomenon, investigating the “what, when, why, and how” of this phenomenon can provide valuable guidelines to educators and policymakers. It is pertinent for students to be motivated to engage in online assessment. Otherwise, they will not attempt to overcome the difficulties and complete the assessments.

Existing quantitative studies related to online assessment during the pandemic provide little insight into the students’ motivation and its importance in online assessment (Khan and Jawaid, 2020; Rahim, 2020). Most studies relied on surveys by using different statements to test a particular model. While these studies ensure many responses and hypotheses testing, they do not capture the nuances afforded by qualitative studies. Tam (2022) calls for more qualitative studies to better understand assessment from the students’ point of view.

Also, there has been excessive focus on instructional design, technological, and cognitive aspects of learning with little attention given to emotional aspects (Montero and Suhonen, 2014). If studies are not focused on learners’ experience, limited knowledge is gained about the role of students’ feelings, needs, and preferences in their online assessments. Failing to explore such pertinent issues “can only offer an incomplete view of the learning experience” (Montero and Suhonen, 2014, p. 165).

The objectives of this study are 2-fold: (1) to examine students’ motivation related to online assessment and (2) to put forth the pedagogical implications of an effective online assessment. The findings of this study may inform educators and policymakers in higher education institutions to address the concerns of students and fine-tune assessment initiatives to facilitate students’ learning and improve learning outcomes. Our research can contribute to the development of the online assessment, for it adds to the current literature by providing empirical evidence in understanding students’ motivation in completing online assessments, especially in the rarely explored context of Malaysia, Lithuania, and Spain. The study focuses on the summative assessment that “occurs at the end of the learning process” (Faulconer et al., 2019, p. 1) where marks, grades, or degrees are given to students (Jones, 1996, p. 134) and enables institutions to make a judgment on one’s performance.

The research question for this study is:

1. What motivates the students to complete their online assessment during the COVID-19 pandemic?

## Literature review

According to Hannaford (2015), motivational beliefs can influence student assessment. The expectancy-value theory of motivation is a practical framework that guides the assessment task that appears to enhance motivation (Simpson, 2013). The theory was developed by Atkinson in 1950 and further established by Wigfield in 192. The theory emphasizes that motivation is crucial for students to complete a task and value their efforts (Wigfield and Eccles, 2000). It is pertinent for students to have an expectation of success and commit to a task with some positive values. If one has high expectations of success but does not appreciate the task assigned, one will not value the task. In answering the question, “Why should I do this task?,” the expectancy-value theory draws our attention to four aspects: intrinsic, attainment, utility, and cost values. Intrinsic value refers to the enjoyment of attempting a task. When learners are intrinsically interested, they become more involved in completing the job. Attainment value details the importance of completing the task well, whereas, utility value is the perception of whether the mission is worthwhile or significant in future. Cost value refers to the effort and commitment to accomplish the task. In general, students tend to experience high-cost value when the assessment is time-consuming and stressful. At the same time, students will experience low-cost value when the online assessment reduces workload, probably by sharing workloads with other students and benefiting from each other.

## Review of related studies on online assessment during the COVID-19 pandemic

An exponential number of papers on the COVID-19 pandemic and learning have been published in recent years (e.g., Guangul et al., 2020; Hasan and Bao, 2020; Scull et al., 2020; Annamalai, 2021; Annamalai et al., 2021; Anh, 2022; Yan-Li et al., 2022). These studies attest to how extensively and immensely the research on the COVID-19 pandemic has proliferated. Some studies focus on students’ learning experience (Hasan and Bao, 2020), educators’ readiness (Annamalai, 2021), teacher stress (MacIntyre et al., 2020), innovation in teaching (Scull et al., 2020; Annamalai et al., 2021), and distance learning during the pandemic (Schneider and Council, 2020).

Guangul et al. (2020) surveyed remote assessment. They identified challenges related to the commitment of the students to submit their review, dishonesty, infrastructure, and assessment of learning outcomes that have been taught. The study suggested that preparing different sets of questions and various types of evaluations will help solve some of the challenges. Sharadgah and Sa’di (2020) examined the preparedness of faculty members for online assessment. The study reported that the educators were not convinced that the online assessment had effectively assessed all the intended learning outcomes. There were also concerns about the lack of experience. It was concluded that many educators are not qualified to conduct the online assessment and they urge higher institutions to consider online invigilation software. White (2020) reported that students hold complex perceptions around their attitudes toward academic integrity and rationalizations of misconduct.

Though studies on online assessment and the COVID-19 pandemic are gaining momentum, it is still unclear whether students are motivated to complete their online assessments during the pandemic. Studies on online assessment during the pandemic remain an understudied area in Malaysia, Spain, and Lithuania. To fill this gap, the study examined students’ motivation in completing their online assessment during the COVID-19 pandemic in the context of Malaysia, Spain, and Lithuania.

## Methodology

This study employed a qualitative research methodology to gain an in-depth understanding of the event in a real-world setting. A phenomenological approach was chosen as it enables the understanding of the phenomenon of assessment through the eyes of students who are experiencing it. In other words, the attempt was to reveal “What’s it like for them?” (Van der Mescht, 2004). Epistemologically, we believe that the phenomenon is socially constructed and given meaning by the individuals experiencing it [see Easterby-Smith et al. (2012)]. The current study used inductive analysis, moving from the concrete to the abstract phases and focusing on non-linear processes that transpired in a natural setting (Lichtman, 2014).

### Participants

A total of 18 students (i.e., six from each country) were selected based on a purposive sampling approach but they were recruited conveniently. The six participants from each country were considered for informational purposes rather than statistical deliberations, as recommended by Lincoln and Guba (1985). More importantly, the engagement with the six participants from each country was sufficient for the saturation point, as evidenced by repeated themes. They were third-year students; therefore, they already had a considerable amount of learning experiences to compare the pre–COVID-19 situation and the situation during the lockdown. All the participants were briefed on the nature of the study, confidentiality, and anonymity. The participants were full-time students from various degree programs related to social science. Their fields of study were education, management, counseling, and language. The participants had never experienced online assessment. Before the COVID-19 pandemic, their assessments were often conducted face-to-face in traditional classroom settings.

### Data collection and analysis

Semi-structured interviews were conducted with the participants during the COVID-19 *via* Webex or MS Teams. The interviews lasted for 40–60 min for each participant. The semi-structured nature of the discussions allowed the interviewer and participants to explore and discuss issues related to online assessment. As argued by Gilbert et al. (2007), interviews may provide richer insights into the phenomenon being investigated and answers to “why” and “how” questions (p. 571).

The interviews were conducted in the student’s native languages—Spanish, Lithuanian, and Malay—and later transcribed verbatim. Translations of the transcriptions into English were done by the three researchers (native speakers of each language) working at the respective universities where the study took place. Two main questions guided the interviews:

1. Can you share your experience related to online assessment during the COVID-19 pandemic?

2. Were you motivated to do your online assessment during the COVID-19 pandemic? Why?

Thematic analysis was employed in this study in analyzing the data. The six-step thematic analysis procedure is: (1) becoming familiar with the data and transcribing all data; (2) generating codes; (3) classifying codes into themes; (4) reviewing and refining themes; (5) concisely defining and naming themes; (6) producing a report from the emerging themes which is a descriptive, analytical, and argumentative narrative. Direct quotations from the participants were included to explain critical themes (Braun and Clarke, 2006, p. 87).

In data familiarization stage, the most common words used were identified. Participants’ keywords and main ideas were considered in generating the initial codes. For example, “dissatisfaction” with submission (L3), “not good” assessment (S5), and “cheating” (L4). These keywords were then converted to the code of inefficient assessments. The initial codes were examined repeatedly and categorized according to similar characteristics to search for themes. The themes were checked to see if the initial codes fit into the classification category. The classification categories were defined and given names indicating their distinct characteristics.

### Trustworthiness in qualitative data

The current study was guided by Guba and Lincoln’s (1994) four qualitative research criteria: confirmability, credibility, dependability, and transferability. Member checking was used to achieve the concept of credibility (Creswell and Poth, 2017). The transcribed interviews were returned to the participants to determine whether the information provided during the interview was the same as the information in the processed data. A description of the setting and participants enabled transferability. When three experienced lecturers coded the themes, investigator triangulation was used. They were able to reach 90% agreement among the coders. As a result, the findings are trustworthy, convincing, and accurately reflect the actual situation. A panel of experts in technology and educational research also validated the interview questions.

## Findings

In presenting the qualitative data, participants from Malaysia were identified as M1, M2, and M3, while participants from Lithuania were L1, L2, L3, and from Spain as S1, S2, and S3.

### Flexibility during the assessment

Majority of the participants felt that the “*assessment suited the whole situation (Pandemic), and everyone adjusted to the new normal of education and the flexibility (e.g., L6).”* Similarly, S3 opined that *it is “good that education did not stop because of the lockdown”* (S3). S2 found that the “*assessment was easier than the one conducted under the normal conditions”* (S2). Students were happy because they “*do not have to commute to the exam hall for 90 minutes exam and can sit for exams via laptop and anywhere they want*” (M3). In fact *“some students study even more during the pandemic”* (S6). Students felt *“calm to sit for the examination in the comfort of their home, in their study rooms, without disturbance from other people*” (S6). L3 liked “*the series of quizzes because revision can be done from the feedback”* (L3) and “*the home environment made [him] calmer in a way, at least in some of the examinations.”* As for L6, he *“thinks professors should make students more comfortable at the beginning of the exams, reassuring students not to worry about potential technical issues.”*

According to M2, the flexibility of the time allowed him to *“research on the questions and understand what is required and it would be clearer for [him] and it would give [him] a whole perspective.”* The flexibility in the time allowed them *“to go over the material once again”* (L5). Similarly, M1 expressed that *“the online assessment was less stressful during the pandemic as students were at home and could manage the time division between work and study.”* Interestingly, M4 elaborated, “if the pandemic occurred 20 years ago, students would be sitting at home *wondering what to do. Luckily it was 2020, and they have technology tools like Zoom, Google Meet, Microsoft Team, and Skype to communicate with their lecturers.”* S6 interestingly pointed out that because of the flexibility of time, her lecturer was able to interact with her; *“she was on maternity leave at that time, but she was commenting on the drafts that I sent to her.”*

**FIGURE 1:**
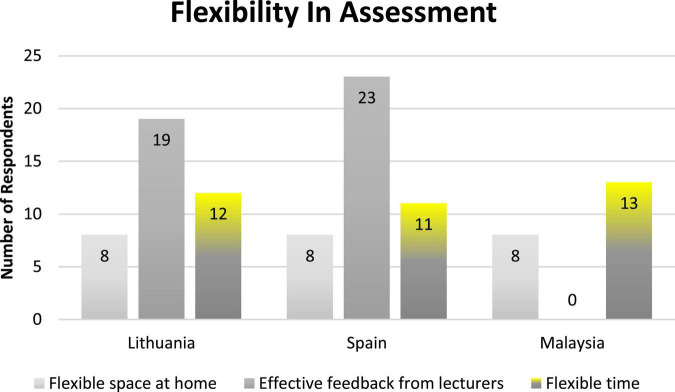
Number of the participants and categories contributing to the theme of flexibility in assessment.

Students were also contented when they *“received feedback from their professors and allowed them to modify and continue working for better grades.”* (S3). The teachers’ feedback also helped them “*to complete their subsequent assignment according to their explanations*” (L7), and they were *“able to understand the topic much better”* (M10). Figure 1 shows the number of participants and categories for the theme related to flexibility in assessment.

### Effective assessment guidelines

Some students felt that it was easier to have an online assessment since more guidance was received compared to the face-to-face assessment. The sudden transition to the online assessment made the lecturers guide the students thoroughly. The lecturers gave *a “rubric as a guide because there were many assignments. The rubrics are very useful because it helps students to understand the reasons behind assessment and marks”* (S4*).* With the rubrics, students *“know why students are given certain marks and not the other.”* (S4). It is practical to have assessment rubrics because *“I was constantly checking the rubrics for doing each part of the work appropriately, for instance, for completing the theoretical part of the assignments, I checked the rubric to see what had already been done and what had yet to be done”* (S1). Rubrics gave them the idea of *what was expected from [them] and how [they] would be assessed* (S1). Students felt that the university had revamped the assessment and they *“benefitted from it because it helped [them] to pass the subjects”* (S2). For example, *reducing the weight of 40 per cent of the final examination mark so that marks from other assignments can take a bigger percentage* (S2).

**FIGURE 2:**
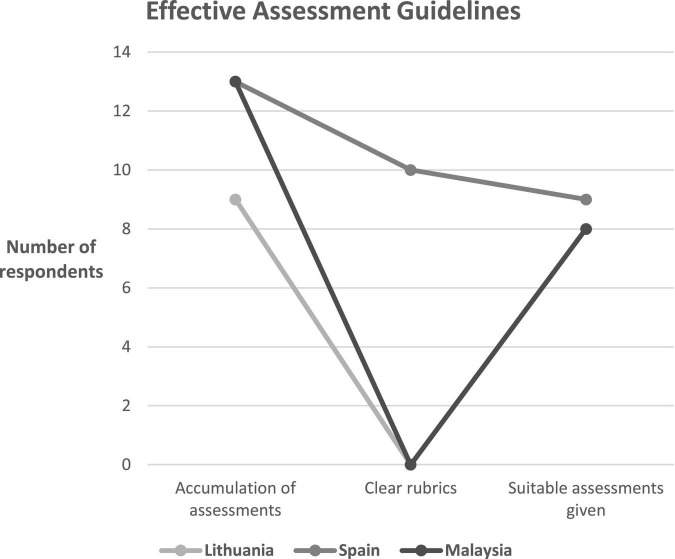
Number of participants and categories contributing to the theme of effective assessment guidelines.

S6 highlighted that *“there were many assignments and different professors in the subject, and each part had its share in the final mark.”* Similarly, L5 said that *“some professors gave quite many tasks for assessment” and “seven separate essays were done in group work.”* Suitable assignments were given to students during the precarious time. Students found that the *“assessments were fun and more participation of students was generated during this pandemic”* (M2). Also, there was a *“requirement to make the assessment criteria more flexible; this helped students achieve better grades”* (S5). Figure 2 shows the distribution of the number of participants who contribute to the theme of “effective assessment guidelines.”

### Stress and fear of the online assessment

The theme of stress and fear was evident in most of the interviews. The online assessment was stressful for L3 because “*many people were living in the house, and [his] grandparents also came to live with [him], and [they] had to share rooms, the spaces for studying, doing assignments and the exams.”* There was also a situation where students needed *“to agree on who uses the computers depending on [their] classes, assignments, mobile data”* (S5), and *“this has affected the assessment and participation in classes”* (S5). The stress was also caused by *“limited time set during the assessment”* (L4) *and “seeing how much time left”* (L3). Most students were stressed *“when the time was spent solving the technical problem”* (L4). The worst tension was caused by the *“fear of disruption of the Internet connection and by losing time for answering the questions* (S2).” L6 expressed her dissatisfaction and detailed that:

*I did not like the oral part of the examination because I have always experienced much more stress, and now, when we do everything online, the fear is immense. It is mainly because of the Internet connection problem.*

**FIGURE 3:**
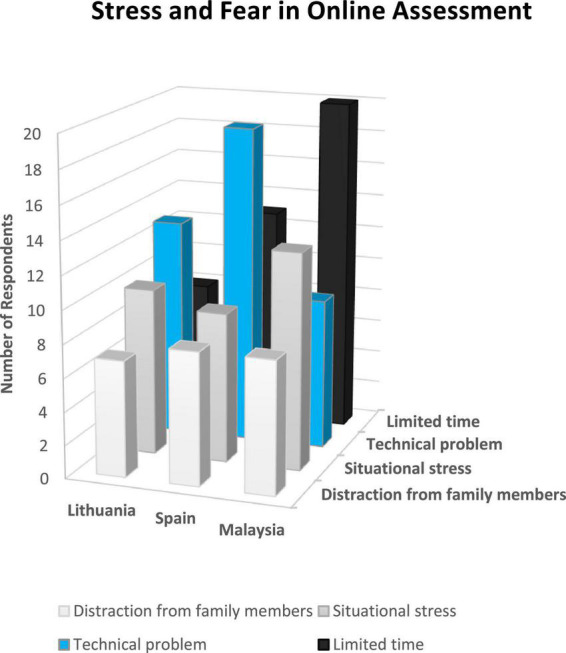
Number of participants and categories contributing to the theme related to stress and fear in online assessment.

According to M5, “*the laptop [she] was using gets heated up during the assessment when used for a long time and reduces the processor speed. This causes the system to lag.”* One student compared the online assessment and highlighted the advantage of classroom assessment. In her words:

*It is less stressful to present in class because even if there is a problem with the slides or something else, you can continue with your presentation. At the same time, you can get cut off entirely during an online production* (L2). Figure 3 shows the number of participants and categories which contribute to the theme related to stress and fear in online assessment.

### Inefficient assessment

Lecturers focus on preventing the students from cheating rather than re-thinking their assessment methods and exam tasks (L6, S2, S5). Therefore, lecturers limited the time for the assessment. This led to *“stress levels for those who work slower, like me”* (L5) and before the pandemic, “*I had a very similar subject with the same lecturer. We wrote the examination in class, and for the writing, part was given an hour and a half”* (L5).

When confronted, the lecturer *“explained their decision and expressed the fear for students to cheat while writing from home when they have more time”* (S2). One student *“submitted a complaint to the Dean’s office about the unfair time limit. She was unhappy about the teacher’s decision to set a limit for answering a question in Moodle.”* L4 said, *“I liked writing when we were given more time, for example, one week or two for essay writing, not just one hour as in the exam.”* (L4). L1 also commented that:

*The time was short for 30 tasks. We had only 15 minutes. It was impossible to concentrate on the tasks because students were thinking about how much time was left, not about making tasks correctly.*

M2 gave a similar account and gave the example of *“an Accounting subject that is usually for three hours and shortened to two hours during the online assessment, thus creating panic whereas three hours is the proper designated time needed to answer the questions as the answers need lengthy and thorough working.”* Participants highlighted that there was *“no standardisation during the online assessment and felt it was all chaos and there was not a single moment when [they] knew for sure what [they] had to do for some of the subjects (S2).”* There were instances where the lecturer *“added new requirements after the assignment was submitted”* (L2). Sl added that *“coordination from the professors should be planned so that the deadlines would not overlap because this stressed [them] a lot”* and *some initial instructions that the students must follow to complete their assessment successfully* (L2*).* S2 lamented, “*I was spending all my time on a diversity of assignments, resulting in less time for studying.*” A student said they *“sit in front of the computer too long because some professors gave quite many tasks for assessment, and [she] was exhausted and waiting for the quarantine to end”* (L1). There was a situation when the students *“have back-to-back exams, and there is no time to revise and remember important points.”* M2 felt “*it was ridiculous to answer nine essay questions from various subjects and eight separate online MCQ questions in six days.*” Similarly, M5 said that *“the idea of a fixed date and time to answer questions from various places cause a problem, and the university should be aware of problems faced by the students.”*

Although students have highlighted the advantages of a flexible environment for assessment, some participants were not satisfied with such evaluations and highlighted the possibilities for students to cheat. For example, *“it could have been done by another person, their father, their mother”* (S6*).* Furthermore, *“the changes in the percentage for examination and when the exam is 40 per cent, and there is no minimum score to pass, it results in that everyone passes without acquiring the knowledge that has to be acquired”* (S6). One technique that the participant disliked was the instrument in the assessment. In S6’s words:

*Every student had to submit in the same folder on Moodle. The deadline was set. And the teacher pointed out that they would review the works if submitted in advance and tell what could be improved before the deadline. Some students presented their papers in advance and were expecting some reflection. However, the teacher reviewed the works only after the deadline passed and expressed their dissatisfaction with some of them. The students were upset because they could have worked on their papers earlier had the teacher reviewed them.*

L3 laments that she *“was informed by the defence committee to look straight in the camera more, but [she] was more focused on the PowerPoint presentation so that [she] could follow what [she] was going to tell next and to explain things.”* She emphasizes that if it was *done “face-to-face, [she] would have indicated at the PowerPoint and would have looked at the committee members more. It is tough to present the Final Thesis orally, to look in the camera and not at the slides.”*

As for S5, one of the lecturers *“used a chat in the forum as a type of participation.”* S5 noticed that *“it was not a good form of assessment, because people repeat what the first or second students have written and write something like “I agree with XXXX or XXXX” without adding anything new. [She] don’t think this is a good assessment tool. But [she] had no doubts that [she] did not like the examination.”* (S5). L4 concurred in this respect and explained her dissatisfaction:

*Sometimes students got lower marks and no explanation was given. A good friend said she tried to prove to the teacher that she did not plagiarise. The teacher said that she checked our work with software and returned it to the student. But the student said she was just reading things online, and maybe that was why the ideas were similar and the sentences were identical.*

As for L1, presentations were complicated because of the *“requirements given by the lecturers. For example, some of the lecturers required students to have their cameras on. If the student has no such opportunity, professors made their grades lower, even if the presentation is well prepared.”* L2 felt comfortable presenting in class for assessment because “*it is much easier to control [her] hands and maintain eye contact.”*

**FIGURE 4:**
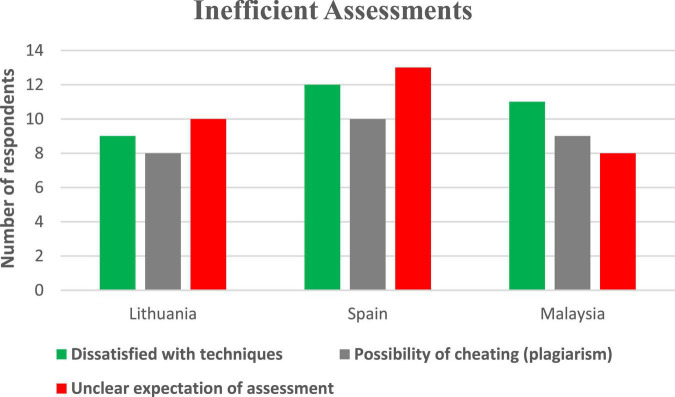
Number of participants and categories for the theme related to inefficient assessment.

M1 was disappointed when the percentage for assessment changed due to the pandemic. M1 explained that *“the lecturer decided to change the portion of the project work which was 10% before the pandemic and now it is said to be 20% since the project work is completed. The percentage for the online assessment will be much lower, which will affect [his] final grade. If [he]knew that the contribution was higher, [he] would have put in the extra effort.”* M3 commented on the *“one-time click assessment, and after some time when students realised the mistakes and wanted to change, they are not able to do so. Students can still rub the wrong answers if the exam is in the classroom.”* Figure 4 illustrates the number of participants and categories for the theme related to inefficient assessment.

## Discussion

Adopting the perspective of the expectancy-value theory of motivation, the interview findings are discussed based on the four main domains of the theory: attainment, intrinsic, cost, and utility values. The participants confirmed that the accumulation of grades for the assessment, clear rubrics, and suitable assessments motivated them to complete their online assessment. These aspects are related to attainment values. With clear rubrics, students could identify expectations and standards for a particular assessment. This, in a way, motivates students to improve their grades. According to Tam (2022), educators’ guidance in explaining various types of examination questions help learners in preparation for their assessment. As revealed by the students, their teachers tend to be more emphatic and sensitive in carrying out online assessments. They seem to understand that students may be temporarily unavailable during the pandemic due to health issues, having other responsibilities such as taking care of their family members, having limited access to technology tools etcetera. In such a situation, Doucet et al. (2020, p. 8) proposed the concept of “Maslow before Bloom” indicating that while teaching and learning activities are important, health and safety must take precedence during a crisis. Nguyen et al. (2020) assert that switching to frequent assessment helps students manage their time wisely and break up their study time into smaller segments. Such periodic assessment gives a lower stake and lowers the stress environment. Incorporating various assessments encourages learners to change their approach from traditional assessment to deep and proactive learning (Jacobs, 2021). The findings also support Felder and Brent’s (2003) idea that assessment supports learning and leads to constructive educational changes. For example, the project work, online presentation, and task-based education evident in the findings allow student-centered learning and active learning to occur.

The domain of intrinsic value is related to flexibility in assessment. The subcategories related to this theme are flexible time, flexible space, and effective feedback from lecturers. The students appreciate the effective feedback from lecturers, encouraging them to promote and maintain their motivation (Benson and Brack, 2010), since well-developed feedback can assist learners who face learning difficulties (Wang, 2014). Students at University S in this study received clear rubrics, but students at Universities L and M did not. Also, feedback from the lecturers was experienced by students from Universities S and L. However, students in University M were not provided with feedback. Regardless, the students were motivated to complete the assessment since they had flexible time and space to complete the assessment. The “space” in which students learn can affect their emotions and engagements (Kayumova and Tippins, 2016). Technology tools resolved the lack of physical attendance, and students were more comfortable and such an environment increased learning outcomes, as affirmed by Goh and Sandars (2020) and Khan and Jawaid (2020). These experiences led students to positive attitudes toward online assessment.

High-cost value was identified when students were demotivated because they experienced distraction from family members, situational stress, technical problems, and limited time to complete the assessment. This is consistent with Tam (2022, p. 12), who argues that “time and technical constraints are inappropriate ways of encouraging students to the fullest with the topics and the assessment process but impose overwhelming pressures on students.” It is also evident from the interview that students were not consulted when changes were made in the percentage of the carry marks for the assessment. Some assessments were also scheduled simultaneously for various subjects (asynchronous online assessment) resulting in students experiencing stress and not performing well in their assessments. Hence, the training of educators is an essential component that institutions of higher learning can no longer depreciate but need to be more rigorous, robust, and advanced. As Boud and Dochy (2010) postulate, assessment practices significantly enhance and enrich university students’ learning experiences because the improvement in assessment practices impacts the quality of students’ learning. Practitioners and educators should have related knowledge, skills, and short-term and long-term planning strategies.

**FIGURE 5:**
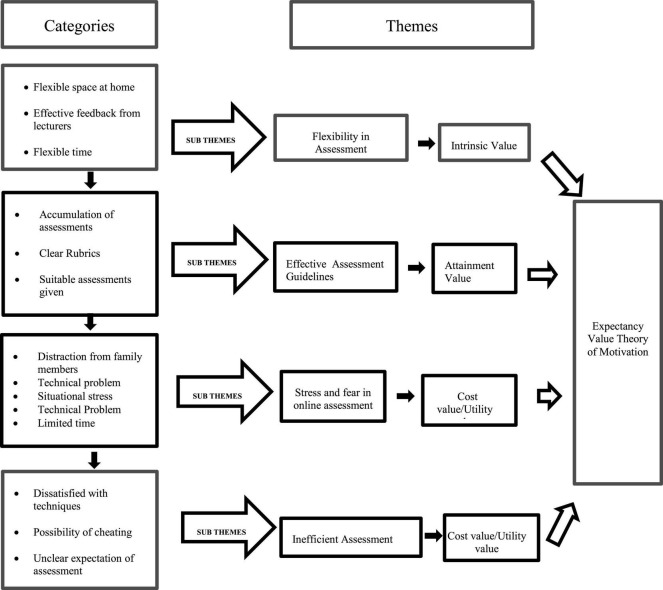
Categories and themes related to online assessment.

The theme related to utility value is not evident in the findings. Since the COVID-19 pandemic has changed the assessment design (techniques of assessment, level of difficulties, students learning strategies and preparation for assessment), learners should be informed on how such assessment can prepare them for future challenges. For example, the importance of communication, collaboration, critical thinking, and creativity should be explained to the students for them to realize the significance of such assessment. According to Gibbs (2010), it is through assessment design that educators create a healthy learning milieu. At the same time, the students also interpreted the online assessment negatively where they felt that online assessment is not very valuable to them because of their dissatisfaction with the techniques, the possibility of cheating among students, and inconsistent assessment expectations. Therefore, there is an overlap between cost value and utility value. Figure 5 illustrates the categories and themes related to online assessment examined in this study.

### Pedagogical implications

The shift to online assessment due to the COVID-19 pandemic is something unexpected. There are challenges and there is no one-size-fits-all approach for the online assessment. However, the findings from the three countries put forth in this study could guide educators in planning their online assessments. Understanding the problems and considering the appropriate strategies are essential to maintain quality in assessment.

Five recommendations can be made from this study, which are:

1. A checklist should be given to teachers and students to support and mitigate the limitations of an online assessment. To develop the list, educators should consult the students and consider the strengths and regulations they will experience during the online assessment. More importantly, in designing the checklist there should be a discussion and negotiation between educators and students so that consensus can be reached. A checklist detailing how the assessment is beneficial to the students should be included to emphasize utility value. The checklist should also include rubrics and feedback.
2. Mock assessment should be conducted to make students aware of the difficulties and consequently work together to overcome the challenges.
3. A discussion on alternate assessments can be arranged if students face technical difficulties. There must always be “Plan B” when the prepared online assessment fails. Plan B will only be executed when students provide evidence (screenshot of technical problems) to avoid dishonesty.
4. Video demos on how assessment could/should be conducted ought to be created to guide students. The video may increase students’ acceptance of online assessments as they would be accustomed to the format and thus have/gain confidence in the system.
5. A well-planned schedule should be created to avoid overlaps in assessment. Some of the assessments are conducted for more than a week (asynchronous online assessment) and it overlaps with other subjects.

## Conclusion

The COVID-19 pandemic has opened venues for online assessment with a completely new experience for educators and learners. This study reveals several significant findings related to assessment and motivation. The use of the virtual environment for online assessment comes with advantages and disadvantages. It is a must for students, instructors, and e-learning policymakers to consider the potential benefits and challenges when encouraging e-learning assessment. Future studies should explore and further investigate and experiment with effective online assessment practices that would contribute to students’ productive and meaningful learning, especially in times of distress, tragedies, and pandemics, as we are currently experiencing. Also, similar comparative studies involving more countries (developed or developing) should be initiated. The themes derived from this study could also be used to develop constructs (and eventually a questionnaire) that is comprehensive and complete to understand online teaching and learning from both the educators’ and the students’ perspectives. The third surge of COVID-19 worldwide means that, most likely, the online assessment may last longer than expected. It also means that teaching and learning in higher institutions should be brave to explore, create, develop, implement, and evaluate new assessment modes that would be feasible for educators and students.

## Data availability statement

The original contributions presented in this study are included in the article/supplementary material, further inquiries can be directed to the corresponding author.

## Ethics statement

Ethical review and approval was not required for the study on human participants in accordance with the local legislation and institutional requirements. Written informed consent for participation was not required for this study in accordance with the national legislation and the institutional requirements.

## Author contributions

All authors listed have made a substantial, direct, and intellectual contribution to the work, and approved it for publication.
